# Stapokibart’s clinical effectiveness in treating moderate-to-severe bullous pemphigoid in the elderly

**DOI:** 10.3389/fimmu.2026.1689991

**Published:** 2026-04-23

**Authors:** Na Du, Yixi Wang, Jingyi Yang, Xinyan Lv, Wei Min

**Affiliations:** 1 Department of Dermatology, The First Affiliated Hospital of Soochow University, Suzhou, China; 2 Department of Dermatology, First People’s Hospital of Kunshan, Suzhou, China

**Keywords:** autoimmune herpetic disease, biologics, bullous pemphigoid, eldly patients, stapokibart

## Abstract

**Objective:**

Bullous pemphigoid (BP) is the most common autoimmune blistering disease in the elderly, for whom conventional systemic corticosteroids and immunosuppressants pose significant safety risks due to age-related comorbidities, leading to infections, osteoporosis, and metabolic disorders. While stapokibart, a novel IL-4Rα inhibitor, holds promise for type 2 inflammatory diseases, clinical evidence regarding its efficacy in BP is currently lacking. Therefore, this study aims to observe the clinical efficacy and long-term safety of stapokibart in the treatment of moderate-to-severe BP in the elderly.

**Methods:**

This single-center, retrospective, uncontrolled case series included 15 elderly patients with moderate-to-severe BP. In addition to conventional topical glucocorticoids or combined systemic therapy, patients received an initial dose of 600 mg stapokibart, followed by 300 mg every 2 weeks. Dosage adjustments were made based on patients’ clinical responses after 16 weeks. The core treatment period was 24 weeks, with a subsequent post-treatment follow-up duration ranging from 4 to 34 weeks (mean 18.78 ± 11.54 weeks). The primary outcome was explicitly defined as the achievement of disease control at 4 weeks. Secondary outcomes included the BP disease area index (BPDAI), dermatology life quality index (DLQI), itch numerical rating scale (NRS), absolute eosinophil count (EO), anti-BP180 antibody levels, relapse rate, and safety profiles, which were assessed at baseline and weeks 2, 4, 8, 16, and 24.

**Results:**

The primary outcome of disease control was achieved in 100.00% (15/15) of the patients within 4 weeks, with a mean time to control of 10.18 ± 3.06 days. Secondary outcomes including BPDAI, DLQI, itch NRS scores, EOs, and anti-BP180 antibody levels were all significantly reduced at week 24 compared to baseline (all *P* < 0.05). During the study and a subsequent mean follow-up duration of 18.78 ± 11.54 weeks, the disease recurrence rate was 26.67% (4/15). Regarding safety, injection site reactions occurred in 13.3% (2/15) of the patients, these were mild and self-limiting, with no patients discontinuing treatment due to adverse events.

**Conclusion:**

Stapokibart may serve as an effective and well-tolerated adjunctive option for alleviating skin lesions and pruritus in elderly patients with moderate-to-severe BP. However, given the small sample size and uncontrolled retrospective design, larger, prospective randomized controlled trials are required to definitively confirm its long-term efficacy and safety.

## Introduction

Autoimmune bullous diseases are a group of rare disorders characterized by pathogenic autoantibodies directed against structural proteins of the skin and mucous membranes. Based on blister depth, these diseases are divided into two main groups. Pemphigus diseases cause intraepithelial blisters, while pemphigoid diseases cause subepithelial blisters (1). Among these, bullous pemphigoid (BP) is a distinct subepithelial autoimmune blistering disorder that predominantly affects the elderly. In recent years, the incidence of BP has been rising, correlating with an aging population and an increase in associated comorbidities (2, 3). The primary clinical manifestation of BP is the development of tense blisters across the body, typically arising on normal skin or edematous erythema, accompanied by varying degrees of pruritus. Beyond the debilitating cutaneous symptoms, BP imposes a substantial clinical and economic burden. The disease is inherently associated with a high mortality rate and an elevated risk of severe systemic infections. Traditional treatment plans include the use of systemic and local corticosteroids, as well as immunosuppressive medications. However, the majority of elderly patients cannot tolerate long-term systemic corticosteroids or other immunosuppressants, as they are typically frail and frequently present with multiple underlying comorbidities. Prolonged use frequently leads to severe steroid-related complications, including profound immunosuppression, worsening of metabolic disorders, and increased susceptibility to potentially fatal infections. These profound clinical challenges strongly justify the urgent need for novel, safer, and targeted therapeutic alternatives.

As the pathophysiological mechanisms are further elucidated, increased expression of helper T-cell 2 (Th2) cytokines, including interleukin-4 (IL-4), IL-13, and IL-5, has been detected in the blood, blister fluid, and skin tissues of BP patients (4), indicating a tight relationship between the etiology of BP and the type 2 inflammatory response (5). Recently, targeting the type 2 inflammatory axis has emerged as a promising therapeutic strategy. Dupilumab, a well-known monoclonal antibody targeting the IL-4 receptor alpha subunit (IL-4Rα), which simultaneously inhibits IL-4 and IL-13 signaling pathways, has accumulated growing clinical evidence demonstrating its ability to suppress type 2 inflammation, alleviate severe pruritus, and halt disease progression in BP (6, 7). Although stapokibart, a novel recombinant humanized IL-4Rα inhibitor, shares a comparable mechanism of action, data on its application in BP remain scarce. Specifically, real-world evidence and long-term safety profiles are critically lacking for frail, elderly patients with comorbidities. To bridge this knowledge gap, this study aims to evaluate the real-world clinical efficacy and safety profile of stapokibart in elderly patients with moderate-to-severe BP, thereby exploring its potential as a steroid-sparing alternative.

## Materials and methods

### Study design and patients

A single-center, retrospective, observational case series was conducted at the Department of Dermatology, The First Affiliated Hospital of Soochow University. Using a consecutive sampling method, we retrospectively collected and analyzed the clinical data of 15 elderly patients diagnosed with moderate-to-severe BP who were treated with stapokibart between September 2024 and February 2025. All patients consecutively admitted during this period who met the eligibility criteria were included in the analysis. The study protocol was reviewed and approved by the Ethics Committee of The First Affiliated Hospital of Soochow University (Approval No.: 2025585).

Inclusion criteria: (i) Individuals with BP diagnosed in compliance with the “Expert Consensus on the Diagnosis and Treatment of bullous pemphigoid (2025)” (8), including histopathology, elevated levels of anti-BP180 and/or anti-BP230 antibodies, distinctive direct immunofluorescence (DIF) or indirect immunofluorescence (IIF) manifestations, and typical clinical manifestations; (ii) Age ≥ 65 years; (iii) An informed consent form was given to the subjects, who willingly signed it after being informed.

Exclusion criteria: (i) Individuals with additional immune system disorders or deficits; (ii) Prior administration of stapokibart for the aforementioned and additional indications; (iii) Any additional biologic agent treatment within six months; (iv) Drug-induced BP (ascertained by thoroughly asking the patient about their past medication use); (v) Any further disorders for which the researcher feels the individual is not eligible to participate in the experiment, such as mental illness, severe hepatic and renal insufficiency, or serious cardio-cerebral and hematological diseases; (vi) Individuals allergies to the active ingredient of stapokibart or any other ingredient.

Criteria for dropout: (i) stopping stapokibart treatment for eight weeks or longer; (ii) not finishing the full twenty-four weeks of treatment or not providing medical history information.

### Intervention protocol

All patients received stapokibart (300 mg/vial) as an adjunctive therapy. Given the current absence of BP-specific dosing guidelines, the stapokibart regimen was based on established protocol for atopic dermatitis. Patients received an initial subcutaneous loading dose of 600 mg in lesion-free areas, followed by a maintenance dose of 300 mg every 2 weeks for the first 16 weeks. At week 16, the dosing interval was extended to 300 mg every 4 weeks if the patient achieved a mild BPDAI score and anti-BP180 antibody levels decreased to less than 1 RU/mL; otherwise, biweekly dosing continued.

### Clinical outcomes and measurement time points

Clinical and laboratory assessments were conducted at baseline (week 0) and at weeks 2, 4, 8, 16, and 24.

Primary outcome: The primary outcome was the disease control rate at 4 weeks following the first dose of stapokibart. Disease control was precisely defined as the healing of preexisting lesions with the absence of new lesions and pruritus.

Secondary outcomes: Secondary outcomes were evaluated at the specified time points and included: (i) BP Disease Area Index (BPDAI) (9): Used to assess disease severity (scored 0–360; ≤ 19 defined as mild, 20–56 as moderate, and ≥ 57 as severe); (ii) Dermatology Life Quality Index (DLQI): Evaluated the impact on the patient’s quality of life (scored 0–30); (iii) Itch Numerical Rating Scale (NRS) (9): Measured peak pruritus over the past 24 hours on a 0–10 scale (0 = no itch, 10 = worst imaginable itch); (iv) Biomarkers: Absolute eosinophil count (EO; normal range 0.02–0.52 × 10^^9^/L) and anti-BP180 antibody levels (normal range 0–20 RU/mL), measured via enzyme-linked immunosorbent assay (OMON Medical Laboratory Diagnostics AG).

Relapse and safety assessment: In patients who achieved initial disease control, clinical relapse/flare was defined as the appearance of ≥ 3 new lesions (blisters or eczema-like/urticaria-like plaques), extensive eczema-like rashes (≥ 10 cm), enlargement of existing lesions, or the return of daily pruritus (10). Safety was assessed continuously by monitoring adverse events, particularly injection site reactions and allergic conjunctivitis, throughout the 24-week treatment phase and the subsequent follow-up period.

### Processing statistics

The patients’ baseline characteristics were described using descriptive statistics. Frequency and percentage were used to describe categorical variables, while x ± s was used to represent continuous variables. One-way repeated measures ANOVA was used to compare data from three or more time points; if *P* > 0.05, the Mauchly sphericity assumption was met and no adjustment was required; if *P* < 0.05, the Greenhouse-Geisser ϵ correction coefficient was applied. The data were processed and analyzed using SPSS 26.0 software, and a difference was considered statistically significant if P < 0.05.

## Results

### Overview

Since there were no shedding cases and there were 15 patients in total, there were still 15 genuine cases that were included in the data statistics, including 6 female and 9 male cases. Between the ages of 67 and 90, the mean age was 80.42 ± 7.90 years; between the ages of 67 and 88, the mean age of diagnosis was 79.58 ± 7.88 years; the disease lasted between 0.12 and 3.5 years, with a mean of 1.05 ± 1.28 years; 9 cases (60.00%) were primary cases and 6 cases (40.00%) were recurrent cases. 3 (20.00%) of the 15 patients acquired hypertension with heart damage, 2 (13.33%) had hyperlipidemia, and 10 (66.67%) had primary hypertension, 8 (53.33%) had diabetes mellitus, 5 (33.33%) had hypoalbuminemia and 1 had coronary artery disease (6.67%). All patients (100.00%) received topical strong glucocorticosteroids at the time of stapokibart application; 10 (66.67%) also received systemic glucocorticosteroid application, 2 (13.33%) received methotrexate, 1 (6.66%) received azathioprine, and 8 (53.33%) received concurrent minocycline, as indicated in **Table 1**.

**Table 1:** Baseline demographic and clinical characteristics of the patients.

Stapokibart group, n = 15		Number
Age (Year, mean ± SD)		72.08 ± 12.62
Diagnostic age (Year, mean ± SD)		79.58 ± 7.88
Disease duration (Year, mean ± SD)		1.05 ± 1.28
Sex, n (%)	Male	9 (60.00%)
	Female	6 (40.00%)
Involvement areas, n (%)	Trunk	15 (100%)
	Extremity	14 (93.33%)
	Oral mucosa	2 (13.33%)
	Scalp	6 (40%)
Comorbidities, n (%)	Hypertension	10 (66.67%)
	Diabetes mellitus	8 (53.33%)
	Hyperlipidemia	2 (13.33%)
	CAD	1 (6.67%)
	Hypoalbuminemia	5 (33.33%)
Related glucocorticosteroid usage (mg/d, mean ± SD)	Maximum steroid dosage before treatment	55.2 ± 15.7
	Steroid dosage just before the treatment	31.7 ± 14.7
	Minimum steroid dosage after the treatment	6.14 ± 2.8
Immunosuppressants before stapokibart, n (%)	Corticosteroids	15 (100%)
	Azathioprine	1 (6.67%)
	Minocycline	8 (53.33%)
	Methotrexate	2 (13.33%)
Concomitant immunosuppressive treatments^†^, n (%)		0 (0.00%)
Prognosis	Disease control time (d) after stapokibart treatment^*^, mean ± SD	10.18 ± 3.06

CAD, coronary artery disease; SD, standard deviation.

^†^None of these treatments had just been started with stapokibart; All of them have been used before.

^*^No patient had the drug discontinued due to treatment failure or adverse reactions.

### Rate of disease control

**Figure 1:**
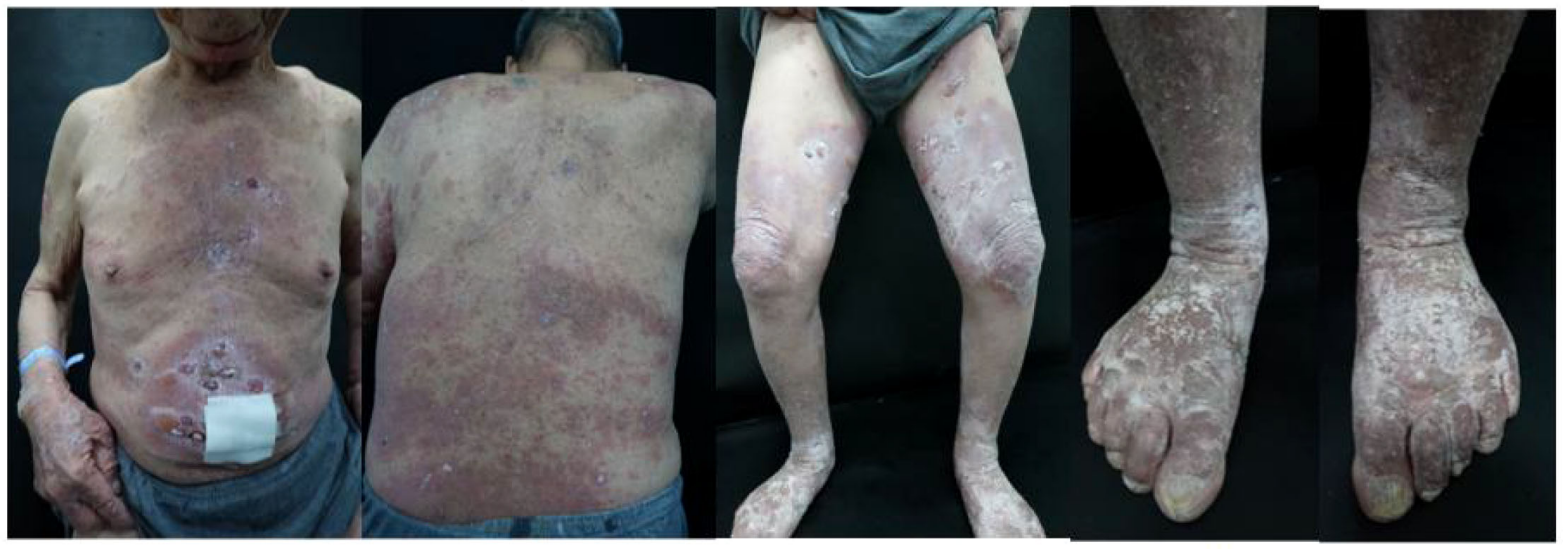
The lesions of patients before treatment.

**Figure 2:**
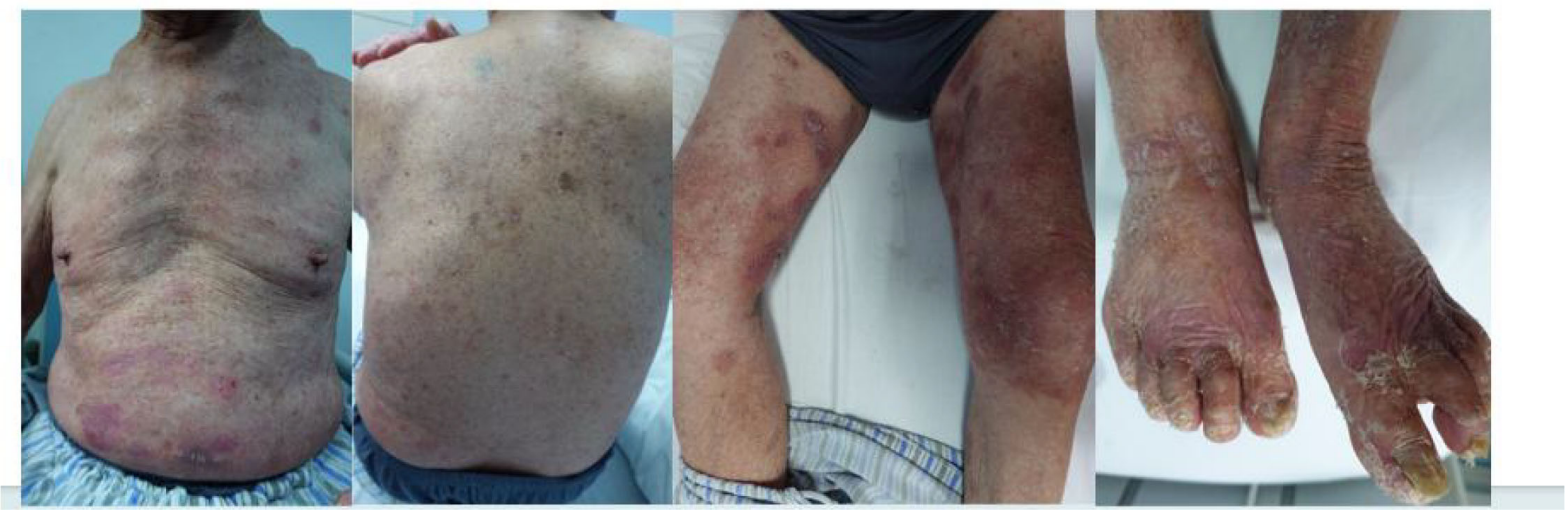
The lesions of patients after 4 weeks treatment of stapokibart.

In 15 cases (100.00%), disease control was attained after 4 weeks of using Stapokibart; in 13 cases (86.67%), disease control was attained within 2 weeks. The mean time to attain illness control was 10.18 ± 3.06 days, with a range of 7 to 14 days. **Figures 1**, **2** illustrate how the patients’ lower back, chest, belly, and both lower limbs showed a reduction in edematous erythema, blisters, and macules following 4 weeks of treatment.

Comparison of BPDAI, DLQI, NRS, EO, and anti-BP180 antibody levels before and after treatment. As indicated in **Table 2** and **Figures 1**, **2**, BPDAI, DLQI, and itching NRS were all lower (*P* < 0.05) following treatment with stapokibart monotherapy (Weeks 2, 4, 8, 16, and 24) than the pre-treatment levels. The DLQI scores of the patients at 8 and 16 weeks of treatment tended to be higher than those at the previous time point, but the difference was not statistically significant. At 4 weeks of treatment, BPDAI and DLQI were lower than those at 2 weeks of treatment (*P* < 0.05); at 16 weeks of treatment, BPDAI and itchy NRS were higher than those at 8 weeks of treatment, but the difference was not statistically significant. Although the difference was not statistically significant, patients’ EO at weeks 2, 4, and 24 was lower than their pre-treatment EO, and their EO at weeks 8 and 16 was also lower than their pre-treatment EO (*P* < 0.05). At the same time, their post-treatment EO at weeks 8 and 16 was higher than their pre-treatment EO, but this difference was not statistically significant either. Anti-BP180 antibody levels were higher in patients at 2 weeks post-treatment than they were before treatment; this difference was not statistically significant, but it did show a declining trend. As indicated in **Table 2**, anti-BP180 antibody levels were lower at 24 weeks post-treatment than before treatment (*P* < 0.05).

**Table 2:** Comparison of BPDAI,DLQI,itching NRS,EO and anti-BP180 antibodies between before and after treatment in 15 patients with bullous pemphigoid (x ± s).

Item	0 week	2 week	4 week	8 week	16 week	24 week
BPDAI	78.80 ± 24.56	11.98 ± 4.50^a^	5.35 ± 3.73^a^^b^	5.28 ± 5.69^a^	10.24 ± 14.86^a^	6.18 ± 7.69^a^
DLQI	22.59 ± 1.97	9.38 ± 2.69^a^	4.42 ± 1.80^a^^b^	5.62 ± 5.60^a^	5.81 ± 7.28^a^	3.21 ± 3.98^a^
Itching NRS	7.43 ± 1.33^a^	2.01 ± 0.73^a^	1.42 ± 0.91^a^	1.42 ± 1.51^a^	2.03 ± 2.80^a^	1.21 ± 1.34^a^
EO(×10^9^/L)	0.71 ± 0.33	0.15 ± 0.78^a^	0.17 ± 0.15^a^	0.28 ± 0.44	0.28 ± 0.48	0.14 ± 0.14^a^
Anti-BP180 antibodies (RU/mL)	185.39 ± 86.98	211.72 ± 130.89	116.22 ± 95.30^b^	74.20 ± 71.02^a^^b^	54.30 ± 83.65^a^	64.98 ± 71.09^a^

Same indexes were compared with those before treatment:

^a^ *P* < 0.05;same indexes were compared with those last obseration time:

^b^ *P* < 0.05; BPDAI indicated bullous pemphigoid diease area index; DLQI indicated dermatology life qulity index;NRS indicated numeical rating scale; EO indicated eosinophil count.

### Rate of disease recurrence

Four patients who were able to control their condition, had a recurrence during the research period. 2 patients occurred at week 8 and week 16 after the treatment, with moderate BPDAI scores; 1 patient occurred at week 8 and week 24 after the treatment, with mild BPDAI scores; and 1 patient occurred at the week 16 following the first treatment, with a severe BPDAI score.

### Negative consequences

After receiving 600 mg of stapokibart for the first time throughout the study, 2 patients experienced moderate pruritic erythema (less than 2 cm in diameter) at the injection site 30 minutes later. This went away on its own in two days. None of the 15 patients with a full record of efficacy assessment experienced any further major adverse events, and none of them stopped taking the medication because of side effects.

After following a 24-week course of treatment, 15 patients were monitored for 4–34 weeks, with an average of 18.78 ± 11.54 weeks. By the 24-week of treatment, all test indices were back to normal, and 14 patients (60.00%) had no skin lesions that had reappeared. With no new erythematous blisters, essentially no itching, and normal eosinophil counts in the retest, along with a negative anti-BP180 antibody, the condition of these 14 patients was stabilized by the 16th week of the follow-up. Stapokibart was then administered to extend the administration time to 300 mg subcutaneous injection every 8 weeks, and the condition has remained stable without recurrence since the prolongation. The follow-up period (24 weeks after treatment ended) showed no recurrence of skin lesions in the other 4 patients who had relapsed during the treatment period, and none of the patients experienced any notable adverse effects.

## Discussion

This study evaluated the clinical efficacy and safety of stapokibart in elderly patients with moderate-to-severe BP. Our findings demonstrate that stapokibart rapidly induced disease control, with 100% of the patients achieving control within 4 weeks (mean time: 10.18 ± 3.06 days). Furthermore, the integration of stapokibart exhibited a substantial steroid-sparing effect, allowing the minimum systemic corticosteroid dosage to be successfully tapered to an average of 6.14 mg/d. These clinical improvements were accompanied by significant reductions in BPDAI, DLQI, and pruritus NRS scores, alongside decreased EOs and anti-BP180 antibody levels. The treatment maintained an acceptable safety profile, with no severe adverse events necessitating drug discontinuation. Regarding long-term outcomes, although mild-to-moderate recurrence was observed in 4 out of 15 patients (26.67%), the majority of the cohort achieved stable remission and maintained clinical responses during the prolonged follow-up period on an extended 8-week dosing interval.

The clinical importance of these findings is particularly pronounced given the demographic most affected by BP. BP is an autoimmune subepidermal dermatosis that predominantly affects older adults (2, 3), a population characterized by distinct clinical vulnerability. Patients with BP are frequently characterized by itchy skin, EO infiltration in the lesions, and high peripheral blood EO counts (11, 12). As observed in our cohort, elderly patients, mean age of 80.42 years, with a high prevalence of underlying conditions such as diabetes mellitus, hypertension, and cardiovascular diseases, are inherently fragile. Glucocorticoids, the cornerstone of BP treatment, can cause severe adverse effects with long-term or high-dose use, particularly in elderly patients with multiple comorbidities, leading to steroid-related complications, such as infections, osteoporosis, metabolic disorders and increased risk of mortality. Consequently, there is an urgent need for safer, long-term therapeutic options that can mitigate these treatment-related risks without compromising disease control.

As the pathophysiological mechanisms of BP are thoroughly investigated, an increasing number of studies have discovered that type 2 inflammatory pathways are crucial to the pathophysiology of BP. Th2 cells and Th2 cytokines, including IL-4 and IL-13, were reported to be markedly elevated in the skin lesions and serum of BP patients in investigations (13). Significant itching is common in BP sufferers as well. Chronic pruritus can occur as a result of IL-4 and IL-13 binding to IL-4R, which can directly stimulate human sensory neurons (14). Furthermore, the type 2 inflammatory response is characterized by higher peripheral blood EO counts and serum levels of total IgE in BP patients (15). Accordingly, when administered to BP patients, the IL-4Rα-targeting biologic dupilumab can considerably alleviate their symptoms (16). Similarly, stapokibart is a humanized monoclonal antibody against IL-4Rα that has been approved for the treatment of type 2 inflammatory diseases, including atopic dermatitis (17–19), severe eosinophilic chronic rhinosinusitis with nasal polyps (20), seasonal allergic rhinitis (21), and others. By binding to IL-4Rα, it can block IL-4 and IL-13 signaling. Additionally, stapokibart binds to IL-4Rα closer to the ligand-binding site than dupilumab, has distinct binding epitopes, and varies in cross-reactivity between species. Stapokibart demonstrates an equal or higher capacity to block IL-4Rα-mediated signaling *in vitro* than dupilumab (22). Translating these findings to routine practice, the primary clinical advantage of stapokibart is its steroid-sparing effect (21). By enabling early and rapid tapering of systemic corticosteroids, it helps clinicians balance disease control with the critical need to limit iatrogenic toxicity. It is well established that high-dose systemic steroids are a major driver of opportunistic infections and mortality in elderly BP cohorts (23, 24). Consequently, minimizing steroid exposure directly translates to fewer fatal complications and a better-preserved quality of life. For frail older patients or those with multiple comorbidities who are poor candidates for conventional immunosuppression, biologics like stapokibart provide a practical and safer strategy for long-term maintenance (25).

The IL-4Rα inhibitor stapokibart is a new potential treatment option for BP, according to the Chinese Expert Consensus on the Diagnosis and Treatment of Herpetiform Pemphigoid Sclerosis (2025). However, there aren’t many extensive clinical trials on the safety and effectiveness of Stapokibart for BP in China. Given the foregoing context, we investigated the use of Stapokibart in the management of moderate-to-severe BP in the elderly.

Within two weeks of starting stapokibart monotherapy, the severity of the condition in our study’s BP patients improved considerably, and by week 4, it had further diminished. As early as one week, patients with BP receiving stapokibart monotherapy showed a significant improvement in new blisters, pruritus, and EO levels without experiencing any notable side effects. Elderly people made up the study population, and most of them had severe conditions with several comorbidities. Infections and hypoproteinemia are frequently linked to severe BP in the elderly, which can significantly affect their psychological health and quality of life. The combination of stapokibart with conventional therapy offers a favorable safety profile, a quick onset of action, and significant long-term efficacy in improving BP. Therefore, we think that stapokibart can assist in inducing and maintaining remission and long-term control when administered as an adjuvant to glucocorticoid tapering or other basal treatments. Enhancing the quality of survival and compliance is beneficial and is anticipated to increase the longevity of older people. Regarding long-term disease control, clinical recurrence were observed in 26.67% (4/15) of our cohort. This recurrence rate is consistent with existing real-world observations for dupilumab in BP (26, 27). Several factors may contribute to these recurrences. Clinically, rapid tapering of systemic steroids can easily expose ongoing, low-level disease activity (27). Pharmacologically, extending the stapokibart dosing interval might cause drug levels to drop below the therapeutic threshold in certain patients. Furthermore, while IL-4Rα blockade safely suppresses inflammation, it does not directly clear long-lived plasma cells or tissue-bound anti-BP180 antibodies, allowing a temporary window for new blisters to form (28, 29). These findings suggest that standard tapering protocols may not suit everyone. Patients with high baseline autoantibodies may require slower steroid tapering and customized biologic dosing intervals (30). Therefore, long-term follow-up is essential to manage late-onset recurrence and determine the most effective maintenance strategy for each individual.

In conclusion, stapokibart may serve as an effective and well-tolerated adjunctive option for alleviating skin lesions and pruritus in elderly patients with moderate-to-severe BP. However, the stability of the results and the level of evidence may be impacted by this single-center, small-sample, uncontrolled study and the dosage of stapokibart in this study was based on its experience in treating atopic dermatitis. Future larger, prospective randomized controlled trials are required to definitively confirm its long-term efficacy and safety in elderly BP patients who do not respond well to systemic therapy or who are intolerant of or contraindicated for systemic therapy.

## Data Availability

The raw data supporting the conclusions of this article will be made available by the authors, without undue reservation.
